# High Prevalence of the *BIM* Deletion Polymorphism in Young Female Breast Cancer in an East Asian Country

**DOI:** 10.1371/journal.pone.0124908

**Published:** 2015-04-24

**Authors:** Ching-Hung Lin, Chen-Yang Shen, Jih-Hsiang Lee, Chiun-Sheng Huang, Chih-Hsin Yang, Wen-Hung Kuo, Dwan-Ying Chang, Chia-Ni Hsiung, Kuan-Ting Kuo, Wei-Wu Chen, I-Chun Chen, Pei-Fang Wu, Sung-Hsin Kuo, Chien-Jen Chen, Yen-Shen Lu, Ann-Lii Cheng

**Affiliations:** 1 Department of Oncology, National Taiwan University Hospital, Taipei, Taiwan; 2 Internal Medicine, National Taiwan University Hospital, Taipei, Taiwan; 3 Graduate Institute of Life Sciences, National Defense Medical Center, Taipei, Taiwan; 4 Institute of Biomedical Sciences, Academia Sinica, Taipei, Taiwan; 5 School of Public Health, China Medical University, Taichung, Taiwan; 6 Department of Surgery, National Taiwan University Hospital, Taipei, Taiwan; 7 Graduate Institute of Oncology and Cancer Research Centre, College of Medicine, National Taiwan University, Taipei, Taiwan; 8 Department of Pathology, National Taiwan University Hospital, Taipei, Taiwan; 9 Genomics Research Center, Academia Sinica, Taipei, Taiwan; The University of Hong Kong, CHINA

## Abstract

**Background:**

A rapid surge of female breast cancer has been observed in young women in several East Asian countries. The *BIM* deletion polymorphism, which confers cell resistance to apoptosis, was recently found exclusively in East Asian people with prevalence rate of 12%. We aimed to evaluate the possible role of this genetic alteration in carcinogenesis of breast cancer in East Asians.

**Method:**

Female healthy volunteers (n = 307), patients in one consecutive stage I-III breast cancer cohort (n = 692) and one metastatic breast cancer cohort (n = 189) were evaluated. *BIM* wild-type and deletion alleles were separately genotyped in genomic DNAs.

**Results:**

Both cancer cohorts consistently showed inverse associations between the *BIM* deletion polymorphism and patient age (≤35 y vs. 36-50 y vs. >50 y: 29% vs. 22% vs. 15%, *P* = 0.006 in the consecutive cohort, and 40% vs. 23% vs. 13%, *P* = 0.023 in the metastatic cohort). In healthy volunteers, the frequencies of the BIM deletion polymorphism were similar (13%-14%) in all age groups. Further analyses indicated that the *BIM* deletion polymorphism was not associated with specific clinicopathologic features, but it was associated with poor overall survival (adjusted hazard ratio 1.71) in the consecutive cohort.

**Conclusions:**

*BIM* deletion polymorphism may be involved in the tumorigenesis of the early-onset breast cancer among East Asians.

## Introduction

The incidence of breast cancer among Asian women is in general lower than that in Western countries. However, all health statistics indicated that breast cancer has been rapidly increasing in recent decades in East Asia, including Singapore, Korea, Japan, and Taiwan [1–4]. Compared to Caucasian American women, the age-period-cohort analyses consistently revealed a much stronger birth cohort effect on the breast cancer incidence of Singaporean, Japanese, and Taiwanese women [1,3,4]. This strong birth cohort effect correlated directly with a rapid increase in the incidence of early-onset breast cancer in these countries. Intuitively, Westernized lifestyle is thought to be the major cause of this rapidly increasing young female breast cancer (YFBC) in Asia [5]. However, our recent study demonstrated a major discrepancy of molecular subtype distributions between Taiwanese and Caucasian YFBC. In contrast to their Western counterpart, Taiwanese YFBCs are characterized by a luminal A subtype (defined as estrogen receptor [ER] and/or progesterone receptor [PR] positive and human epidermal growth factor receptor 2 [HER2] negative) prevalence, and low basal-like subtype prevalence [6]. These findings implied that the emerging YFBCs in Taiwan might not just be a mirror image of their Western counterparts. We hypothesize that some unique genetic factors or interactions between genetic factors and environmental factors may play a role in East Asian YFBC carcinogenesis.

Recently, Ng KP et al. discovered a common germline polymorphism (deletion of intron 2 of *BIM* gene) which was uniquely detected in East-Asian people (12.3% carrier frequency) and was absent in Africans and Caucasians. *BIM* deletion polymorphism conferred an inferior response to tyrosine kinase inhibitors in patients with chronic myeloid leukemia and epidermal growth factor receptor mutated non-small cell lung cancer [7–9]. The *BIM* gene encodes B-cell lymphoma 2 interacting mediator of cell death (BIM) protein, which is a member of the Bcl-2 family. Wild type BIM protein, which contains a BCL2-homology domain 3 (BH3), which functions as an apoptosis facilitator and has been shown to mediate apoptosis in response to stimuli such as cytokine deprivation, deregulated calcium flux and microtubule perturbation. Thus, BIM is considered a protector of tissue homeostasis [10,11]. The *BIM* deletion polymorphism switched *BIM* splicing from exon 4 to exon 3, and resulted in expression of BIM isoforms lacking the pro-apoptotic BH3 domain and conferred intrinsic resistance to tyrosine kinase inhibitors [7].

Since *BIM* deletion polymorphism is unique in East Asian people, and its product (BIM isoforms) may be linked to tumorigenesis, it is crucial to clarify whether this genetic change plays a role in the carcinogenesis of YFBC in East Asian women.

## Materials and Methods

### Patients and sample collection

All participants in this study gave written informed consent. The study received approval from the National Taiwan University Hospital (NTUH) ethics committee (201307001RINA). The study included 307 female healthy volunteers, 692 patients with stage I-III breast cancer in one consecutive cohort and 189 patients with ER+/HER2- breast cancer in one metastatic cohort with available germline or tumor DNAs (S1–S3 Datasets). The healthy volunteers participated in the prior study exploring the association of breast cancer and gene polymorphism [12]. The consecutive cancer cohort was obtained from a prospectively collected database which included stage I-III breast cancer consecutively newly diagnosed at NTUH between 2004 and 2006 [6]. The metastatic cancer cohort was obtained from a retrospectively collected database which includes patients with ER+/ HER2- metastatic breast cancer patients diagnosed at NTUH between 2001 and 2006. To avoid bias by double counting, we excluded 19 patients from the consecutive cohort because these patients were included in the consecutive cohort and had distant metastasis between 2004 and 2006. The methods and definitions of ER and HER2 positivity were previously described [6]. For ER and PR, Tumors with ≥10% positively-staining nuclei were considered positive. The HER2 status was considered positive if score 3+ by immunohistochemical analysis or 2+ with gene amplification on fluorescence *in situ* hybridization.

### Evaluation of *BIM* deletion polymorphism

The DNAs from healthy volunteers were extracted from blood specimens. The DNAs from patients in consecutive and metastatic cancer cohorts were extracted from formalin-fixed paraffin-embedded tumor specimens. The genomic DNAs of blood and tumor specimens were isolated using the QIAamp DNA Mini Kit (Qiagen Inc., Valencia, CA, USA). For DNAs from each blood or tumor specimen, we performed two separate polymerase chain reaction reactions to determine the presence of the wild-type and deletion alleles as previously described [7]. The forward and reverse primers for the deletion allele were CCACCAATGGAAAAGGTTCA and GGCACAGCCTCTATGGAGAA, respectively. The forward and reverse primers for the wild-type allele were CCACCAATGGAAAAGGTTCA and CTGTCATTTCTCCCCACCAC, respectively. The resulting PCR products from the deletion (1,323 bp) and the wild-type (4,226 bp) alleles were analyzed on 1% agarose gels. For each PCR, we used genomic DNAs from KCL22 and PC-9 cells as positive and negative control, respectively.

### Statistical analysis

Data on clinicopathological features between wild and deleted *BIM* groups were compared using chi-square test (or two tailed Fisher’s exact test if expected number of each cell was less than five cases). The Mantel-Haenszel chi-square test was used to verify age, histologic grade, tumor size, lymph node status and American Joint Committee on Cancer (AJCC) stage-related trend. For survival analysis in the consecutive cancer cohort, only patients with stage I-III breast cancer were included, and distant metastasis free survival (DMFS) and overall survival (OS) were used as the endpoints. DMFS was defined as the duration from diagnosis to confirmation of distant recurrences. OS was defined as the duration from breast cancer diagnosis to death from any cause. Survival curves were constructed using the Kaplan-Meier method. The associations between each of the categorical variables and survival were analyzed using the log-rank test. Cox proportional hazards analysis was used to determine the relative contribution of various factors to survival. The backward stepwise variable selection procedure was applied to obtain the best candidate final Cox’s proportional hazards model. A *P* value ≤0.05 was used to indicate statistical significance; all tests were two-tailed. All statistical analyses were performed using the statistical package SPSS for Windows (Version 17.0).

## Results

### Frequencies of *BIM* deletion polymorphism by age groups among healthy volunteers and breast cancer patients

The healthy volunteers had a median age of 48 (range 25–80) years at enrollment. The patients in consecutive and metastatic cancer cohorts had a median age of 49 (range 23–86) years and 50 (range 26–82) years at initial diagnosis of breast cancer, respectively. Among the 307 healthy volunteers, *BIM* deletion polymorphism was detected in 48 (14%) subjects and the frequencies were similar among the three age groups (≤35 vs. 36–50 vs. >50 years, 13% vs. 14% vs. 14%, *P* = 0.974). Compared with healthy volunteers, patients in the consecutive cancer cohort (19% vs. 14%, P = 0.018) had significantly higher frequencies of *BIM* deletion polymorphism and patients in the metastatic cancer cohort (19% vs. 14%, *P* = 0.089) had a trend toward higher frequencies of *BIM* deletion polymorphism (Table 1).

**Table 1:** Comparison of *BIM* deletion polymorphism in women benign breast disease and two breast cancer cohorts with healthy volunteers among age groups.

	No. (%)											
	≤35 years			36–50 years			>50 years			Whole		
*BIM* polymorphism	wild	deleted	*P*	wild	deleted	*P*	wild	deleted	*P*	wild	deleted	*P*
Healthy (reference)	42 (88)	6 (13)		140 (86)	22 (14)		125 (86)	20 (14)		307 (86)	48 (14)	
Consecutive cohort	37 (71)	15 (29)	0.045	254 (78)	71 (22)	0.029	267 (85)	48 (15)	0.640	558 (81)	134 (19)	0.018
Metastatic cohort	9 (60)	6 (40)	0.028*	62 (78)	18 (23)	0.079	82 (87)	12 (13)	0.820	153 (81)	36 (19)	0.089

* two tailed Fisher’s exact test.

**Fig 1:**
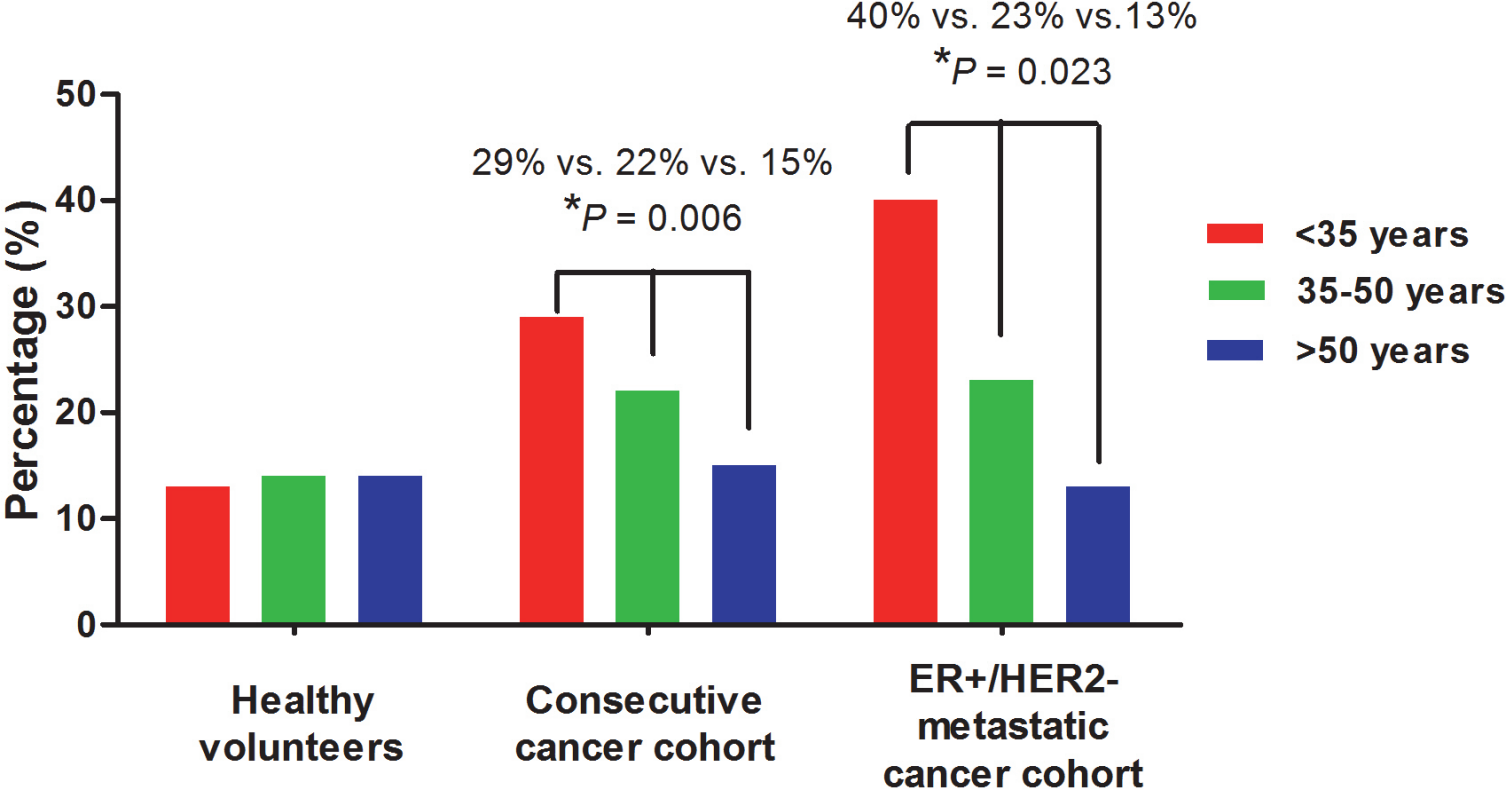
Inverse association of *BIM* deletion polymorphism with age in young breast cancer patients.

Both cancer cohorts consistently showed inverse association of *BIM* deletion polymorphism with patient age (≤35 years vs. 36–50 years vs. >50 years: 29% vs. 22% vs. 15%, *P* = 0.006 in consecutive cohort, and 40% vs. 23% vs. 13%, *P* = 0.023 in metastatic cohort). Among women ≤50 years, both cancer cohorts consistently showed higher frequencies of *BIM* deletion polymorphism than healthy volunteers (consecutive cancer cohort, 23% vs.13%, *P* = 0.005; metastatic cancer cohort, 25% vs. 13%, *P* = 0.010). In contrast, the frequencies of *BIM* deletion polymorphism were quite similar (healthy volunteer, 14%; consecutive cancer cohort, 15%; metastatic cancer cohort, 13%) among the three study groups of women >50 years (Table 1 and Fig 1).

### Patient clinicopathological characteristics by *BIM* status in two breast cancer cohorts

Among the 692 patients with stage I-III breast cancer in the consecutive cohort, *BIM* deletion polymorphism was detected in 140 (19%) subjects. The *BIM* deletion polymorphism rate was significantly higher in young patients. Other clinicopathological variables including histology, histologic grade, AJCC stage, ER status, PR status, HER2 status and molecular subtype were not significantly associated with this polymorphism (Table 2).

**Table 2:** The characteristics of patients in the consecutive cancer cohort by *BIM* deletion polymorphism.

Characteristics	No.	No. (%)		
	All (n = 692)	*BIM* wild (n = 558)	*BIM* deleted (n = 134)	*P* value
Age at initial diagnosis				0.006
≤35 years	52	37 (71)	15 (29)	
36–50 years	325	254 (78)	71 (22)	
>50 years	315	267 (85)	48 (15)	
Histology type				0.783
Ductal carcinoma	659	532 (81)	127 (19)	
Others	33	26 (79)	7 (21)	
Histologic grade				0.556
I	131	108 (82)	23 (18)	
II	362	293 (81)	69 (19)	
III	166	131 (79)	35 (20)	
Unknown	33	26	7	
Tumor size				0.186
<2 cm	300	237 (79)	63 (21)	
2–5 cm	331	268 (81)	68 (19)	
>5 cm	61	53 (87)	8 (13)	
Axillary lymph node				0.494
None or cN0	374	298 (80)	76 (20)	
1–3 or cN1	194	160 (82)	34 (18)	
4–9 or cN2	78	60 (77)	18 (23)	
≥10 or cN3	46	40 (87)	6 (13)	
AJCC stage				0.881
I	237	191 (81)	46 (19)	
II	327	265 (81)	62 (19)	
III	128	102 (80)	26 (20)	
ER status				0.434
Negative	221	182 (82)	39 (18)	
Positive	471	376 (80)	95 (20)	
PR status				0.921
Negative	395	318 (81)	77 (19)	
Positive	297	240 (81)	57 (19)	
HER2 status				0.137
Negative	544	445 (82)	99 (18)	
Positive	148	113 (76)	35 (24)	

AJCC, American Joint Committee on Cancer; ER, estrogen receptor; PR, progesterone receptor; HER2, human epidermal growth factor receptor 2.

Among the 189 patients with stage ER+/HER2- metastatic breast cancer in the metastatic cohort, *BIM* deletion polymorphism was detected in 36 (19%) subjects. Consistent with that in consecutive cohort, the *BIM* deletion polymorphism rate was significantly higher in young patients and clinicopathological variables including histology, histologic grade, PR status, recurrence status and metastatic site were not significantly associated with *BIM* deletion polymorphism (Table 3).

**Table 3:** The characteristics of patients in ER+/ HER2- metastatic cancer cohort by *BIM* deletion polymorphism.

Characteristics	No.	No. (%)		
	All (n = 189)	*BIM* wild (n = 153)	*BIM* deleted (n = 36)	*P* value
Age at initial diagnosis				0.023*
≤35 years	15	9 (60)	6 (40)	
36–50 years	80	62 (78)	18 (23)	
>50 years	94	82 (87)	12 (13)	
Histology				0.374
Ductal carcinoma	162	133 (82)	29 (18)	
Others	19	14 (74)	5 (26)	
Unknown	8	6	2	
Grade				0.583
I	27	22 (81)	5 (19)	
II	75	62 (83)	13 (17)	
III	29	22 (76)	7 (24)	
Unknown	58	47	11	
PR Status				0.139
Negative	61	53 (87)	8 (13)	
Positive	126	98 (78)	28 (22)	
Unknown	2	2	0	

DFI, disease-free interval; ER, estrogen receptor; PR, progesterone receptor; HER2, human epidermal growth factor receptor 2.

*two tailed Fisher’s exact test.

### Prognostic value of *BIM* deletion polymorphism in patients with stage I-III breast cancer

In consecutive cancer cohort, the median follow-up duration among the 692 patients with stage I-III breast cancer was 81.7 months (95% confidence interval (CI), 80.1–83.4). The 6-year distant metastasis-free survival rate was 92% for stage I disease, 82% for stage II disease, and 70% for stage III disease. The 6-year overall survival rate was 95% for stage I disease, 90% for stage II disease, and 74% for stage III disease.

Traditional prognostic factors such as tumor size, axillary lymph node status, ER expression, PR expression and HER2 status were associated with DMFS and/or OS in univariate and/or multivariate analyses. Univariate determined that *BIM* deletion polymorphism was not associated with DMFS (hazard ratio [HR] = 1.11, *P* = 0.636) and OS (HR = 1.45, *P* = 0.125). Multivariate analysis determined that *BIM* deletion polymorphism was not associated with DMFS, but it was significantly associated with shorter OS (adjusted HR = 1.71, *P* = 0.027) (Table 4).

**Table 4:** Analyses of distant metastasis-free and overall survival in patients with stage I-III breast cancer.

		DMFS				OS			
	No.	HR (95% CI)	*P*	Adjusted HR (95% CI)	*P*	HR (95% CI)	*P*	Adjusted HR (95% CI)	*P*
*BIM* polymorphism			0.636		NS		0.125		0.027
Wild	558	1.00				1.00		1.00	
Deleted	134	1.11 (0.72–1.73)				1.45 (0.90–2.33)		1.71 (1.06–2.77)	
Age			0.629		NS		0.008		0.033
≤35 years	52	1.00				1.00		1.00	
36–50 years	325	1.13 (0.51–2.48)				0.53 (0.24–1.16)		0.48 (0.22–1.06)	
>50 years	315	1.32 (0.60–2.88)				1.08 (0.51–2.27)		0.85 (0.40–1.82)	
Histologic grade			0.008		NS		0.002		NS
I	131	1.00				1.00			
II	362	1.82 (01.0–3.31)				1.92 (0.90–4.10)			
III	166	2.80 (1.49–5.25)				3.67 (1.70–7.93)			
Unknown	33	2.75 (1.14–6.64)				3.38 (1.22–9.32)			
Tumor size			<0.001		0.022		<0.001		0.020
≤ 2 cm	300	1.00		1.00		1.00		1.00	
2–5 cm	311	1.81 (1.19–2.74)		1.35 (0.87–2.09)		2.20 (1.32–3.68)		1.49 (0.87–2.56)	
> 5 cm	61	4.34 (2.55–7.38)		2.38 (1.29–4.40)		5.66 (3.09–10.36)		2.69 (1.34–5.41)	
Axillary lymph node			<0.001		<0.001		<0.001		0.001
None or cN0	374	1.00		1.00		1.00		1.00	
1–3 or cN1	194	2.79 (1.81–4.30)		2.47 (1.58–3.89)		2.39 (1.41–4.06)		2.29 (1.31–4.01)	
4–9 or cN2	78	3.04 (1.76–5.26)		2.26 (1.24–4.12)		4.13 (2.29–7.44)		3.26 (1.70–6.23)	
≥ 10 or cN3	46	4.43 (2.46–7.99)		2.97 (1.54–5.72)		5.39 (2.84–10.24)		3.67 (1.78–7.57)	
ER status			0.008		0.004		<0.001		<0.001
Negative	221	1.00		1.00		1.00		1.00	
Positive	471	0.61 (0.42–0.88)		0.58 (0.40–0.84)		0.41 (0.27–0.62)		0.43 (0.28–0.67)	
PR status			0.003		NS		<0.001		NS
Negative	396	1.00				1.00			
Positive	296	0.56 (0.38–0.82)				0.40 (0.25–0.65)			
HER2 status			0.313		NS		0.509		NS
No	544	1.00				1.00			
Yes	148	1.24 (0.82–1.87)				1.18 (0.73–1.90)			

DMFS, distant metastasis-free survival; OS, overall survival; HR, hazard ratio; CI, confidence interval; ER, estrogen receptor; PR, progesterone receptor; HER2, human epidermal growth factor receptor 2

## Discussion

High prevalence of *BIM* deletion polymorphism in young Taiwanese breast cancer patients suggests that this East Asian specific genetic trait is involved in the tumorigenesis of early-onset breast cancer among East Asians. This finding supports our hypothesis that, in addition to environmental and lifestyle factors, certain genetic factors may play a role in Asian young breast cancer development.

*BIM* deletion polymorphism was found exclusively in East Asian individuals (12.3% carrier frequency) [7]. In our study, the frequency of the polymorphism in the whole healthy volunteers (14%) was close to that reported by Ng KP et al., and the frequencies were similar among ≤35 (13%), 36–50 (14%), and >50 (14%) age groups. In breast cancer patients >50 years, the frequencies of *BIM* deletion polymorphism in both cancer cohorts were not significantly different from healthy volunteers. In contrast, among subjects <50 years, higher *BIM* deletion polymorphism frequencies were consistently shown in both cancer cohorts than healthy volunteers. Among very young (≤35 years) patients, the frequencies of *BIM* deletion polymorphism reached up to 29% and 40% in the consecutive and metastatic cancer cohorts, respectively.

Since no significant association between *BIM* deletion polymorphism and other clinicopathological factors except age was observed, we hypothesize that *BIM* deletion polymorphism may mediate the cancer initiation rather than tumor progression. However, how and why this genetic change affects the young ladies in East Asians remains unclear. In addition, the major limitation of the present study is lack of comprehensive information of breast cancer risk factors such as menstruation history, family history, pregnancy and birth history, alcohol consumption, and weight. To confirm *BIM* deletion polymorphism as a susceptible gene for YFBC carcinogenesis in East Asia, the validation by a well designed case control study is mandatory.

BIM is essential for initiation of various physiological apoptotic situations, including developmentally programmed cell death and stress-induced apoptosis. BIM is considered a protector of tissue homeostasis [10,11]. The breakdown of tissue homeostasis may lead to various pathological situations including tumor formation. In the mouse model of B cells and kidney epithelial cells, the loss of single *BIM* sensitizes the mice to tumorigenesis [13,14]. In mammary gland, disruption of the BH3-only proapoptotic factor BIM in mice prevents induction of apoptosis in and clearing of the lumen in terminal end buds during puberty. The findings indicate that BIM is a critical regulator of luminal space formation and maintenance during mammary morphogenesis [15–17]. Since *BIM* deletion polymorphism is germline genetic event and we did not observe significant association between *BIM* deletion polymorphism and other clinicopathological factors except age, we suggest that *BIM* deletion polymorphism may mediate the cancer initiation rather than tumor progression in human mammary gland.

BIM plays important roles not only in tumorigenesis but also in treatment response. Previous preclinical studies have shown that BIM plays a key role in the anoikis, an apoptosis triggered by detachment from the extracellular matrix, of various tumor cells [18–20]. Absence of wild type BIM protein has been linked to resistance to chemotherapy and tyrosine kinase inhibitors in several cancer types [14,21–30]. In breast cancer, decrease of BIM expression has been linked to resistance to estrogen deprivation and a HER2 tyrosine kinase inhibitor [31,32]. *BIM* deletion polymorphism is heterozygous, so it can transcribe both wild type BIM protein and BIM isoforms which lack the BH3 domain. Although wild type BIM protein from single allele may retain certain pro-apoptosis functions, the net activity of BIM protein and isoforms in *BIM* deletion polymorphism cells remains inadequate in certain tumor types [7]. Our multivariate analysis showed that *BIM* deletion polymorphism was significantly associated with shorter OS in patients with stage I-III breast cancer (Table 4). However, it was not significantly associated with DMFS. Because of the association between *BIM* deletion polymorphism and age group, we conducted the stratified survival analysis in the three age groups (≤35, 36–50, and >50 years). Although the association of *BIM* deletion polymorphism with DMFS and OS did not reach statistical significance in the three age groups, the adjusted HRs were numerically higher in younger patients. In age group ≤35 year, the adjusted HRs were 6.03 for DMFS, and 1.99 for OS (S1 Table). Therefore, the prognostic value of *BIM* deletion polymorphism in patients with breast cancer warrants to be validated.

Samples used for genotyping in this study were different between healthy volunteers (blood) and cancer patients (tumor). We have analyzed both the wild and deletion alleles with positive and negative controls and did not detect homozygous deletion of *BIM* gene in any individual sample. In addition, both cancer cohorts consistently showed inverse association of age with *BIM* deletion polymorphism. Among cancer patients, women ≤50 years had significantly higher frequency of *BIM* deletion polymorphism than patients aged >50 years (Fig 1). Therefore, the use of different types of samples is unlikely to produce bias of our findings.

In summary, we have discovered a high prevalence of *BIM* deletion polymorphism in young patients with breast cancer in Taiwan, and this polymorphism may be associated with patients' poor survival. As a potential susceptible genetic factor, *BIM* deletion polymorphism may interact with their contemporary environmental and lifestyle factors and contribute to the YFBC carcinogenesis in East Asians. Clarification of the underlying mechanisms and interaction between *BIM* deletion and their contemporary environmental factors is an important step toward mitigating the rapid surge of YFBC in East Asia.

## Supporting Information

S1 Dataset Raw data of healthy volunteers. (XLS)

S2 Dataset Raw data of patients in the consecutive cohort. (XLS)

S3 Dataset Raw data of patients in the metastatic cohort. (XLS)

S1 Table Stratified survival analyses of *BIM* deletion polymorphism by age groups in patients with stage I-III breast cancer. (DOCX)
