# Supplementary material for: High Prevalence of the BIM Deletion Polymorphism in Young Female Breast Cancer in an East Asian Country
Source: PLoS One. 2015 Apr 24;10(4):e0124908. doi: 10.1371/journal.pone.0124908 (PMC4409392; doi:10.1371/journal.pone.0124908)
Supplement: S1 Table — (DOCX) [file pone.0124908.s004.docx]

## S1 Table. Stratified survival analyses of *BIM* deletion polymorphism by age groups in patients with stage I-III breast cancer

|  |  | **DMFS** | | | | **OS** | | | |
| --- | --- | --- | --- | --- | --- | --- | --- | --- | --- |
|  | **No.** | **HR (95% CI)** | ***P*** | **Adjusted HR (95% CI)** | ***P*** | **HR (95% CI)** | ***P*** | **Adjusted HR (95% CI)** | ***P*** |
| **Whole** |  |  | 0.636 |  | 0.335 |  | 0.125 |  | 0.021 |
| *BIM* wild | 558 | 1.00 |  | 1.00 |  | 1.00 |  | 1.00 |  |
| *BIM* deleted | 134 | 1.11 (0.72-1.73) |  | 1.25 (0.80-1.95 |  | 1.45 (0.90-2.33) |  | 1.76 (1.09-2.85) |  |
| **<35 years** |  |  | 0.136 |  | 0.158 |  | 0.769 |  | 0.532 |
| *BIM* wild | 37 | 1.00 |  | 1.00 |  | 1.00 |  | 1.00 |  |
| *BIM* deleted | 15 | 3.12 (0.70-13.96) |  | 6.03 (0.50-73.27) |  | 1.24 (0.30-5.21) |  | 1.99 (0.23-17.12) |  |
| **36-50 years** |  |  | 0.710 |  | 0.700 |  | 0.216 |  | 0.143 |
| *BIM* wild | 254 | 1.00 |  | 1.00 |  | 1.00 |  | 1.00 |  |
| *BIM* deleted | 71 | 1.13 (0.60-2.10) |  | 1.14 (0.59-2.17) |  | 1.64 (0.75-3.61) |  | 1.92 (0.80-4.58) |  |
| **>50 years** |  |  | 0.837 |  | 0.908 |  | 0.237 |  | 0.168 |
| *BIM* wild | 267 | 1.00 |  | 1.00 |  | 1.00 |  | 1.00 |  |
| *BIM* deleted | 48 | 0.93 (0.44-1.95) |  | 0.96 (0.45-2.05) |  | 1.49 (0.77-2.90) |  | 1.61 (0.82-3.18) |  |

DMFS, distant metastasis-free survival ; OS, overall survival; HR, hazard ratio; CI, confidence interval

The adjusted HRs were obtained by Cox proportional hazards analyses without stepwise selection procedure. The variables in the analyses include *BIM* deletion polymorphism, age group, histologic grade, tumor size, axillary lymph node, ER, PR, and HER2 status.
